# Measuring cough severity: Perspectives from the literature and from patients with chronic cough

**DOI:** 10.1186/1745-9974-5-5

**Published:** 2009-03-19

**Authors:** Margaret Vernon, Nancy Kline Leidy, Alise Nacson, Linda Nelsen

**Affiliations:** 1 United BioSource Corporation, Bethesda, MD, USA; 2 Merck & Co Inc, North Wales, PA, USA

## Abstract

**Background:**

In order to assess severity of cough from patients' perspectives and capture the effects of treatment in clinical trials, a measurement tool must show evidence of validity and reliability. The purpose of this study was to characterize cough severity from patients' perspectives as the initial step in the development of a new patient-reported outcome (PRO) measure for use in clinical trials.

**Methods:**

This focus groups study included patients with clinician confirmed chronic cough recruited from a large internal medicine clinic in the US. A semi-structured focus group guide was designed to elicit information about patients' experiences with cough severity and their characterization of symptoms. The focus group data were coded to identify concepts and terminology of cough severity.

**Results:**

Three focus groups were conducted [n = 22; 6 male; mean age 66.1 (± 12.9)]. Etiology included GERD, asthma, bronchitis, post-nasal drip, and other. Three domains of cough severity were identified: frequency, intensity, and disruption. In addition to a single cough, participants in all focus groups described coughing in uncontrollable paroxysms they called "fits," "bouts," "spells," or "episodes." The urge to cough, described as an important sign of impending cough, was considered a component of cough frequency. Participants also described daytime activity and nighttime sleep disruption as an indication of cough severity. Finally, participants described variability in cough severity.

**Conclusion:**

Results suggest that patients describe cough severity in terms of frequency, intensity, and disruptiveness, indicating these 3 domains should be addressed when evaluating cough severity and outcomes of treatment.

## Introduction

Chronic, persistent cough is a frustrating and bothersome symptom for many adults; loss of sleep, exhaustion, irritability, urinary incontinence, cough syncope, social disability, and inability to perform daily activities are some of the negative outcomes associated with this condition [1]. Many persons experience chronic cough secondary to another medical condition, such as COPD, asthma, rhinosinusitis, GERD, post-nasal drip, or unknown etiology. Chronic cough has been defined as cough present for more than 8 weeks; subacute cough is generally considered to last between 3–8 weeks, and may be the result of unresolved symptoms of respiratory infection [2,3]. Cough is a common symptom and a frequently stated reason for health care visits [2].

When assessing the effectiveness of new therapies for reducing cough symptom severity in a clinical trial setting, patient reported outcomes (PROs) are an important methodological tool for evaluating treatment effectiveness. Whether selecting an existing instrument or developing a new PRO instrument, qualitative research in the form of interviews or focus groups is important to ensure that the content of the instrument is consistent with patients' experiences and that the concepts measured by the instrument include the elements that patients' consider most important about a condition or a treatment intervention in order to enhance measurement precision [4]. Quantitative research is critical to ensuring that the instrument is suitably reliable and valid to be able to detect treatment effects [5].

A recent literature review was conducted to determine PROs that have been used and are publicly available to measure cough symptom severity in chronic and subacute cough patient populations, as well as to evaluate whether any of the instruments uncovered in the review have documented evidence of good psychometric properties [6]. Articles were reviewed that included PRO measures as primary or secondary endpoints in clinical trial settings. In addition, articles were reviewed that provided information surrounding the development or measurement properties of PROs designed to evaluate cough symptom severity. Particular attention was given to the content and design of PRO cough measures (item content, recall period, mode of administration, response scale, response anchors). Included in the literature review were articles that were published in English between 2002 and 2006. Eighteen papers met the review inclusion and exclusion criteria and were reviewed in full.

Three validated instruments were found that evaluate health-related quality of life (HRQL) in cough populations [e.g., the Leicester Cough Questionnaire (LCQ) [7]; the Cough Specific Quality of Life Questionnaire (CQLQ) [8]; and the Chronic Cough Impact Questionnaire (CCIQ)[9]. These questionnaires have multiple items and domains and address the impact of cough on various aspects of health-related quality of life, including physical, psychological, and social domains, and ask patients to reflect on the effect cough has had on their lives. As such, they are useful to capture the *impacts *of cough symptoms, but are not suitable to evaluate the severity of the symptom per se. No validated PRO measures of cough symptom severity were identified within the published literature. Further, no articles detailing qualitative work to support the content of cough symptom severity measures were found. Among the cough symptom severity measures used in clinical trial settings, there were no two symptom severity measures that were exactly alike in content or design.

Content of these instruments provide insight into the aspects of cough severity considered important from a clinical trial perspective. Six of the studies reviewed included a measure that assessed frequency of individual coughs or frequency of 'periods' or 'bouts' of coughing [10-15]. In addition, six studies measured nighttime coughing or sleep disturbance (awakening) due to coughing [10-15]. Several studies used measures that assessed daytime disruption due to coughing (e.g., cannot perform most usual activates due to coughing) [8,10,12-15]. Finally, several studies reviewed included measures that assessed intensity of cough (e.g., distressing cough; chest/abdominal pain) [11-15].

In terms of measurement design, subjective cough symptom items uncovered in the literature review often had 10-point Visual Analogue-type response scales (VAS) with two descriptive terms to anchor the extreme values [7,13,14,16-19]. For example, the phrases "no cough" to "most severe cough" have been used as anchors for severity items, among others. Items to evaluate cough were often administered as pen and paper patient-completed daily diaries with 24-hour recall periods [17].

Findings from the literature review suggested that concepts to consider when evaluating cough severity include both the frequency and intensity of cough. These concepts were often measured daily with a single item using a VAS-type response scale. Although common concepts and design elements were identified, no standard measure is available for capturing cough symptom severity. An instrument based on sound science and the principles of instrument development could result in a tool with greater precision and sensitivity of measurement, providing a better understanding of the magnitude and pattern of cough in a variety of patients, and more accurately capturing the effects of treatment on this important symptom in a clinical trial setting. Because this is a phenomenon experienced by the patient, the instrument should be consistent with patients' descriptions of the important facets of cough symptom severity, as well as the words they use to describe this symptom and their day-to-day cough experiences in order to ensure that the tool has content validity [4]. No qualitative evidence was found in the literature review that indicated that the concepts included in the cough symptom severity items used are consistent with patients' descriptions of symptoms or symptom experience.

The purpose of this study was to evaluate patients' perspectives on cough symptom severity by conducting focus groups with chronic cough patients with the end goal of selecting, adapting, or developing a new cough severity instrument that would be consistent with patients' experiences and thus increase measurement validity, precision, and standardization of cough symptom severity. Specific objectives of the current research were: 1) to gather information about patient perceptions of the attributes of cough, 2) to identify key subjective issues and concerns related to chronic and/or subacute cough severity and 3) to identify the language and terminology patients use to describe their cough, with a focus on cough provocation, intensity, frequency, and periodicity.

## Methods

### Design

This cross-sectional, qualitative study included 3 focus group sessions with participants who had a diagnosis of chronic cough with various etiologies. Participants for the focus groups were recruited by a large internal medicine clinic in suburban Washington, DC. Eligible participants were identified through chart review and were screened to ensure that they met the study entry criteria. They were eligible to participate in the chronic cough group if they had a dry, non-productive cough for ≥ 8 weeks due to asthma, GERD, or some other unknown condition. Participants with moderate to severe COPD were excluded given that cough associated with this condition is often productive and may only be one symptom among other respiratory symptoms. Participants were ineligible if they were currently taking antibiotics, or medications known to cause a cough (e.g., ACE Inhibitors), if they had allergic rhinitis, congestive heart failure, or if they had a current upper respiratory tract infection. Inclusion/exclusion criteria allowed for the recruitment of subacute cough participants in addition to chronic cough participants but no subacute cough participants were enrolled in this study.

### Study Procedures & Focus Group Guide

The focus groups were held at a focus group facility and moderated by researchers trained in qualitative methods and focus group moderation. All participants provided written informed consent prior to the focus group discussions. With the consent of participants, all discussions were audio-recorded and observed by members from the research team through a one-way mirrored window. The moderator followed a pre-scripted semi-structured focus group guide when leading the discussion, and participants were encouraged to discuss their experiences with each other. The focus group guide was designed to elicit information on the patient terminology and experiences specifically related to cough, including descriptions of cough, severity of cough, frequency of cough, cough at night, variability in frequency of cough, concept of episode, sensitivities (triggers of cough), as well as variability in cough severity. Specifically, the group discussions were opened with very general questions (Please describe your cough). As participants began to discuss and describe more specific attributes of their cough, themes that emerged were followed up on with neutral probes using the language that participants used. For example, after the concept of frequency of cough was brought up by at least one person, the group was asked as a whole to describe the frequency of their cough using the terminology that was already spontaneously discussed. After at least one person described coughing at night (and/or disruption caused by nighttime cough), the group was asked to discuss experiences with coughing at night. In this way, the guide started with very general questions and themes and terminology were allowed to emerge organically. The moderator did not introduce words and themes that had not been previously used in the discussion.

The primary purpose of the study was to explore patients' experiences with cough symptom severity, including cough frequency, intensity, provocation, and periodicity. Each session lasted for approximately 90 minutes. At the conclusion of each discussion, participants were asked to complete a brief sociodemographic and clinical questionnaire and were remunerated $50 for their participation.

### Qualitative Analysis Approach

A content analysis approach was used to analyze the data (transcripts, field notes and audio-recordings) from the focus group sessions. The focus groups were audio recorded and transcribed; verbatim transcripts were analyzed using ATLAS.ti qualitative data analysis software [20]. General themes related to cough severity, issues, and concerns about chronic cough and side effects of treatment were identified and coded in the focus group transcripts. Each transcript was independently coded by two researchers, and codes were compared. When differences in coding occurred, codes were reconciled through collaborative review and re-reading of each focus group transcript. Themes that emerged from the data were organized in order to develop a conceptual model of cough severity.

## Results

### Sample

A total of 22 chronic cough participants were included in the focus group discussions. Saturation, or the point at which no substantially new information continues to emerge, was reached for cough symptoms upon completion of the third focus group, thus three focus groups were conducted [4]. The average age of study participants was 66.1 ± 12.9 years, and 72.7% of the sample was female. Among study participants, all were Caucasian and more than half the sample reported a household income greater than $60,000 per year. The most common etiologies of chronic cough as reported by patients were gastroesophageal reflux disease (GERD) (n = 7), asthma (n = 5), bronchitis (n = 4), and post-nasal drip syndrome (n = 4). Other causes of chronic cough reported by patients included irritant exposure, post-infectious cough, and mild chronic obstructive pulmonary disease (COPD). Most participants (86.7%) reported having completed diagnostic tests for their cough.

Descriptive statistics of the sociodemographic and clinical questionnaire showed that almost all of the participants reported coughing while performing daytime activities and all but three participants reported some coughing while trying to sleep at night. All participants had experienced their cough for greater than 8 weeks, and most of the participants (81.8%) reported experiencing their cough for greater than one year and 31.8% of participants rated their cough severity as "very severe;" 50% rated their cough severity as "moderate;" 13.6% considered their cough to be "mild;" and 4.6% considered their cough to be "very mild." With respect to treatment, most of the participants (n = 17) reported taking prescription medication, while others reported the use of over-the-counter treatments (n = 8) and/or herbal or other home remedies (n = 7).

### Focus Group Discussion Results

Review of the focus group transcripts identified the emergence of three major themes which were used to develop the coding dictionary and used in qualitative analysis. First, participants in all groups discussed *triggers of and treatments for cough *at length. For example, participants discussed such triggers as spicy food, air conditioning, and perfumes, all of which were reported to provoke coughing. In addition, participants in each group were eager to discuss a variety of treatment options and homeopathic remedies, including prescription treatments, over-the-counter treatments, and behavioral modifications. While triggers and treatments were extensively discussed, this report does not summarize these topics in-depth as they do not pertain to the description of the attributes of cough severity. Secondly, participants described *the attributes of cough and the characteristics of cough *in terms of both intensity (e.g., deep cough) and frequency (e.g., constantly). Finally, in discussions about cough intensity and frequency, participants often discussed *disruption of daily routines or activities and disruption to nighttime sleep*. In the results sections that follow, attributes and characteristics of cough are discussed first. The final section discusses the quantity and quality of disruption to patients' daily routines and activities as well as disruption of nighttime sleep due to cough. It should be noted that in order to clearly present the information patients provided, major concept themes are discussed separately in the results sections that follow. During the focus groups, concepts and themes that we discuss in separate sections for clarity of presentation were often discussed jointly and were highly related for the participants.

### Description of Cough Intensity

At the beginning of the focus group sessions, participants were asked to describe their cough. A variety of words were offered to describe the intensity of the cough: hacking, deep, strong, harsh, intense, deep, and barking.

When describing experiences with coughing, participants discussed physical discomfort and physical reactions such as pain or vomiting resulting from a particularly intense cough:

...I keep coughing, and that's when your throat starts to hurt.

I will cough, and cough, and cough, and cough until I basically have triggered, you know, trying to vomit in my stomach kind of thing.

I've gotten to that point where I'm coughing, I mean, the diaphragm, the rib cage, umm, is painful.

Participants also offered descriptions of other qualities of their cough, including whether the cough was productive or non-productive. The majority of participants in this sample described their cough as non-productive.

Mine is very hacking, and it's not intense or deep, and nothing comes up. It's not productive.... It's dry hacking.

### The Urge to Cough & the Coughing Episode

When describing their cough, participants also discussed two experiences that were related to coughing but differed from their description of an individual cough: the sensation of having the urge to cough and the experience of having coughing fits, episodes, or bouts. Urge to cough was generally discussed as the antecedent to a cough, and most often described as a tickling sensation in the throat. A coughing fit or episode was described as an uncontrollable bout of coughing lasting for more than one or two individual coughs. These two experiences were highly related to the experience of coughing for participants in these focus groups. Urge to cough was described in the following ways:

When I wake up in the morning, I can feel-if I feel a tickle back in the back of my throat I know ultimately I'll end up coughing during the day at some point or another.

*I get this tickle and I have to cough, you know, and it's all day*.

Many of the participants described having coughing paroxysms, which were termed fits, bouts, or episodes. These episodes were often described in terms of coughing uncontrollably for some duration of time. While there was perhaps some ability to control or fight off of the urge to cough or an individual cough, there was little control over the cough during an episode:

I have it under control with the medication, but I still get some fits that usually last five minutes, and they're rather intense, uh, in coughing.

...I had a coughing jag at work one day that was totally embarrassing. I couldn't stop. ...occasionally I'll have a real episode, but it's more just every once in a while.

...but normally during the day I'll have just attacks of four, five, or six coughs and then it will clear up...

### Frequency of Urge, Cough, and Episode

In addition to discussing the qualities of the urge to cough, the cough, and the episode, participants also discussed the frequency of their urges to cough, coughing, and coughing episodes. For participants in these focus groups, the frequency of the urge, the amount of coughing, and the number of coughing fits or episodes that they experienced on any particular day was related to how 'bad' or severe their coughing was for that day. As one participant noted, "So frequency is an issue and so is how bad, how severe the cough is...so it does fall within some continuum." In addition, participants also discussed how often they experienced coughing during the night. Sample quotations representing this concept are as follows:

*Mine's just all the time. I cough all the time*.

For me it would be intermittent coughing throughout the day.

I will cough, you know, like occasionally, but not continuously unless I get something more severe.

Sometimes I can't sleep at all night. I have to get up and go get in a chair, because whatever is coming out of my head or my throat is sitting in this bronchial tube down there, and it's messing up everything. And it's continuous.

Umm, I pretty much cough all day and all night, but I've taken some medication.

Uh, yes, uh, I wake up at least by midnight and have a series of coughs and then about 3:00.

I'm not coughing at all and, you know, it could be months before I get another cough, but when I get a cough, the frequency is close to ten and how bad it is can be a ten.

While many people describe the frequency of their coughing during the day and at night with descriptive frequency terms (continuous, constantly, intermittent, occasional, a little), participants in all 3 focus groups reported that they could not accurately account for the exact number of coughs that they had over a given time period:

I don't really know how often I cough, 'cause I'm oblivious to it many times. My wife will tell me that I'm on the telephone coughing. So, that's part of probably just being not as aware sometimes as other people would be.

I don't think anyone can tell you how many times they cough a day. You're asking about frequency. I really don't.

And sometimes I'm coughing, I don't even realize it. You know.

Finally, participants also described the frequency of their urge to cough as well as the frequency of episodes:

*...You should just stop coughing. Just stop. Don't do it. And I say but I get this tickle and I have to cough, you know, and it's all day*.

Five, six, seven, eight [episodes]. I mean this is a really good time right now in here.

*It's very infrequent episodes*.

### Variability in Frequency

Many participants reported that the amount of coughing that they experience varies both over the course of one day as well as from day to day. For example, some of the participants reported that they experience more frequent coughing in the morning, some reported more frequent coughing in the afternoon, and some reported that they cough more frequently at night or that the cough comes back at night. However, among this group of participants, there were no clear patterns in terms of when during the day participants were likely to experience more or less coughing. Participants discussed more variability in frequency from day to day than within one day. Participants said things like:

*Some days are...much worse than others*.

No, I think everyday, you know, everyday is different.

*...Some days I don't have anything like that, and all of a sudden I might have one [episode]*.

### Daytime Activity Disruption

Participants anchored the severity of their cough to the disruption it caused, including social disruption that they experience due to their frequent cough, which included reports of being embarrassed in public situations and having concerns that their coughing disturbs others around them. In addition, participants discussed the impact of cough on their emotional state, including feelings of annoyance, irritation, frustration and worry about the implications of the intensity of their cough on their health. Participants also discussed the ways in which their cough disrupted their work, causing them to regularly have to step out of the room during meetings or having difficulty when talking with colleagues. Finally, participants discussed other activity disruption including intense coughing occurring when driving a car, having an episode at a restaurant or while eating, and having to cancel plans because of coughing. Participants had comments such as:

*It's embarrassing sometimes, too. I mean, either you're-if I'm standing in a grocery line and start coughing, people are looking at me like I'm contagious with something*.

But, like I said, I live in fear of that cough and that cough has come back.

I don't cough all the time, but it is embarrassing. You will be in a restaurant, when you start coughing. Really, it's embarrassing.

I was talking to the person that was reporting to me and giving her direction or what to do, that I could not complete the sentence without coughing. And that was so annoying and embarrassing.

### Nighttime Sleep Disruption

For participants who experienced coughing at night or when lying down, disrupted sleep had a particularly debilitating effect on daytime functioning. Participants discussed experiencing sleep disruption due to coughing as well as daytime impacts of this sleep disturbance:

I've also learned to sleep sitting up, so basically sitting up like [inaudible]. I mean, I don't have this every night, but when I'm going through a period where I'm having that, expecting it, I just try to sleep sitting up as much as I can, uh, to avoid getting any – I mean, you move, so you're-then I slide down and I start coughing and then I wake up. So, like you, I often find I don't get a good night's sleep.

...I cough a lot when I go to bed. I lay down, and I find that makes me cough a lot.

I'll cough. Sometimes I can't sleep at all night.

### Overlap in Discussion of Severity Concepts

While the concepts of cough severity identified in the data have been presented separately for clarity, the concepts of frequency, intensity, and disruption were often interconnected for participants. In one utterance, for example, participants could have discussed how their intense cough disrupted their meetings at work or how their frequent nighttime coughing disrupted sleep. Several quotes are presented below to illustrate the interconnectedness of cough severity concepts.

For some, intensity and frequency were interconnected:

But what's intense about it is it keeps on in the-on and on, the days that I'm coughing. Not the amount, er, or the physical thing, and it just exhausts me, and I just get sick to death when one starts and I think, Here we go. And then, you know-but it's, it's mostly the, the pattern of it during the day, versus what some people are describing as you know, it's, it's, like, I'm not gonna break a rib or anything. It's-but it's hacking.

And when I have a cough on those occasions, it's probably close to a ten. I mean, it will just – I mean, it will just continue and it's painful, the rib cage, the diaphragm, and it'll be all day, all night.

For others, intensity and disruption to activities were interconnected:

To me that strikes me as always a very serious bout of coughing, because obviously, oxygen wasn't getting where it was supposed to be getting. Um, and I find this is freaky, especially if you're driving, you know, and your coughing, and all of a sudden you're dizzy.

For others, frequency and disruption to activities were interconnected:

I had one on a job interview, and by the time I was done trying to speak, I sounded like Minnie Mouse on speed. I was so squeaky. But it [the cough] wouldn't stop, just would not stop.

Are you coughing less, are you feeling better, are you sleeping through the night.

For me it's not being able to sleep, you know, just waking up coughing, coughing, and coughing.

Finally, intensity, frequency and disruption can all be interconnected:

And that then trickles down here, and then I cough like the devil... I get-it sticks here, great big chunks of it will stick here-can't get it out. And I cough, and cough, and cough, and then if you go to a restaurant and start coughing, they throw you out.

The final cough was absolutely – absolutely retched and, ugh, I think was XXXXX who mentioned embarrassed. Ugh, I had a lot of – I had a number of meetings that I – that I had to go to and, uh, I couldn't stop coughing in those things.

## Discussion

Results of the focus groups suggest the concept of cough severity is a single concept with three inter-related components: frequency, intensity, and disruption. Frequency included the urge or 'tickle' that preceded coughing. Although technically not a "cough" as defined medically, patients considered this sensation an inseparable part of the cough experience indicating it should be a component of an evaluation tool. Paroxysms of coughing, which were extended bouts of individual coughs and that were described as less controllable than a single cough, were also a component of frequency. Intensity of coughing was described in terms of how 'deep,' 'hard,' or 'harsh,' the coughing was, and intense coughing sometimes had broader physical affects including pain, discomfort, and vomiting. Patients grounded their discussion of severity in terms of daytime and nighttime disruptions due to frequent or intense coughing. For example, daily activities such as work and leisure were disrupted by coughing. Loss of sleep was a particularly debilitating effect of frequent nighttime coughing for participants because coughing at night caused sleep disruption and daytime consequences of sleep disruption. Qualitatively, patients saw disruption as an indicator of cough severity, again indicating this information should be captured in a cough severity tool.

**Figure 1:**
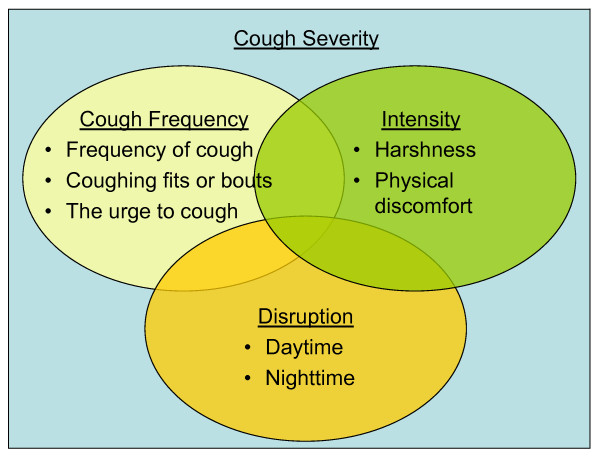
**Conceptual Diagram**. Non-proportional Venn Diagram depicting the interrelationships between the concepts that make up total cough severity including cough frequency, cough intensity, and disruption caused by cough. Overlapping areas are non-proportional and do not represent statistical magnitude of relationships between content domains.

Based on the results above and informed by the literature review, a conceptual diagram showing the proposed measurement concepts of cough severity was developed (See Figure 1). This hypothesized conceptualization of cough severity as a measurement concept will inform the development of a new PRO measure designed to evaluate cough symptom severity.

While the frequency of coughing, urge, and episodes were discussed as one important component of how 'bad' or severe the cough is, participants reported that they would not be able to report the exact number of coughs they had over the course of the day but preferred descriptive terms on a continuum from no coughing to constant coughing. Words in the middle of the continuum, between no coughing and continuously coughing, were described in terms such as intermittent, occasional, moderate, and a little. Participants also reported that they did experience variability from day to day in the severity of coughing. Thus, severity of cough is likely best measured on a daily basis to capture the natural variability in cough severity from day to day.

These findings suggest that a multifaceted symptom severity instrument that measures the domains of cough frequency, intensity, and disruptiveness would provide optimal content coverage given patients' experiences. Given that cough severity items uncovered in the literature review often had 10-point VAS-type response scales, a 0–10 response scale might be optimal for measurement of cough severity indicators. As cough severity may vary from day to day, a daily diary with a 24-hour recall period might best capture natural day-to-day variability in cough severity. It should be noted that this study included a relatively small non-representative sample, and all focus groups were conducted in one region of the United States; thus, results may not be generalizable to more diverse or international samples. Future research will involve the development of an instrument, evaluation of content validity and clarity through cognitive debriefing interviews, and quantitative evaluation of reliability, validity, and responsiveness in larger, more diverse sample sizes. Furthermore, research will be undertaken to determine whether results are generalizable to subacute cough populations.

## Conclusion

The purpose of this study was to gather patient input on the concept of cough severity in order to better understand the patient perspective of this important symptom and inform the selection, adaptation, or development of a cough severity assessment tool to ensure that the content of the tool would be consistent with patients' experiences of cough severity and therefore would be suitably sensitive for use as an efficacy endpoint in clinical trials. Findings suggest cough intensity, frequency, and disruptiveness are important domains of cough severity. Closely related to individual coughs, the frequency of the urge to cough as well as the experience of coughing paroxysms were relevant to discussions of cough severity. Participants anchored severity through descriptions of disruption in daytime activity and nighttime sleep. Finally, participants discussed variability in severity of coughing from day to day – some days the cough was less intense, less frequent, or less disruptive while other days the cough was more severe. Together, this information provides the form and structure for a new patient-reported outcome measure to quantify cough severity.

## Competing interests

Linda Nelsen is an employee of Merck & Co., Inc. Margaret Vernon, Nancy Kline Leidy, and Alise Nacson are consultants to Merck & Co., Inc.

## Authors' contributions

All authors contributed to study design, study implementation, analysis, and writing of the manuscript.
